# Amplified Inhibition of Stellate Cell Activation Pathways by PPAR-γ, RAR and RXR Agonists

**DOI:** 10.1371/journal.pone.0076541

**Published:** 2013-10-01

**Authors:** Efrat Sharvit, Shirley Abramovitch, Shimon Reif, Rafael Bruck

**Affiliations:** 1 Institute of Gastroenterology and Liver Diseases, Tel Aviv Sourasky Medical Center, and Sackler Faculty of Medicine, Tel Aviv, Israel; 2 Pediatric Gastroenterology Unit, Dana Children’s Hospital, Tel Aviv Sourasky Medical Center, Tel Aviv, Israel; 3 Sackler Faculty of Medicine, Tel Aviv University, Tel Aviv, Israel; Haassah Medical Center, Israel

## Abstract

Peroxisome proliferator activator receptors (PPAR) ligands such as 15-Δ12,13-prostaglandin L(2) [PJ] and all trans retinoic acid (ATRA) have been shown to inhibit the development of liver fibrosis. The role of ligands of retinoic X receptor (RXR) and its ligand, 9-*cis*, is less clear. The purpose of this study was to investigate the effects of combined treatment of the three ligends, PJ, ATRA and 9-*cis*, on key events during liver fibrosis in rat primary hepatic stellate cells (HSCs). We found that the anti-proliferative effect of the combined treatment of PJ, ATRA and 9-*cis* on HSCs was additive. Further experiments revealed that this inhibition was due to cell cycle arrest at the G_0_/G_1_ phase as demonstrated by FACS analysis. In addition, the combined treatment reduced cyclin D1 expression and increased p21 and p27 protein levels. Furthermore, we found that the three ligands down regulated the phosphorylation of mTOR and p70^S6K^. The activation of HSCs was also inhibited by the three ligands as shown by inhibition of vitamin A lipid droplets depletion from HSCs. Studies using real time PCR and western blot analysis showed marked inhibition of collagen Iα1 and αSMA by the combination of the three ligands. These findings suggest that the combined use of PJ, ATRA and 9-*cis* causes inhibition of cell proliferation by cell cycle arrest and down-regulation of fibrotic markers to a greater extent compared to each of the ligands alone.

## Introduction

Hepatic fibrosis is a pathological response of the liver to acute and chronic insults such as viral infection, cholestasis, toxins and metabolic diseases. Hepatic stellate cells (HSCs) play a crucial role in liver fibrosis. HSCs are responsible for excessive deposition of extracellular matrix (ECM) proteins.

The nuclear superfamily of hormone receptors includes peroxisome-proliferator activated receptor γ (PPARγ), retinoic acid receptor (RAR) and vitamin D receptor (VDR). These receptors are transcription factors that regulate transcription of a variety of genes. RAR and PPARγ hetrodimerize with the retinoic X receptor (RXR) and affect transcriptional activation of target genes [1].

PPARγ plays a key role in HSCs biology and is involved in the maintenance of a quiescent HSCs phenotype [2]. PPARγ inhibits AP-1 and profibrogenic gene expression and activation of HSCs results in loss of PPARγ inhibition [3]. Treatment of HSCs with synthetic PPARγ ligands suppresses the fibrogenetic potential of HSCs *in vitro* and *in vivo* [3-5].

It has been shown that 15-Δ12,13-prostaglandin L(2) [PJ], a PPARγ ligand, inhibited cell proliferation and led to cell cycle arrest in HSCs cell line [6] and inhibited ECM expression [4,7]. All-trans retinoic acid (ATRA), a RAR ligand, inhibited the expression of procollagen I, III, IV, fibronectin and laminin, α Smooth muscle actin (αSMA), transforming growth factor (TGF-β) and IL-6, but had no effect on HSCs proliferation [8,9]. The RXR ligand, 9-*cis*, had a different effect on HSCs: 9-*cis* inhibited HSCs proliferation but increased procollagen I mRNA and had no effect on other ECM proteins [8].

Although each of the ligands has an effect on liver fibrosis, these effects are minor. We previous demonstrated that rats with hepatic fibrosis that were treated with PPARγ and RAR agonist led to additive inhibitory effect on proliferation and to reduced expression of TGF-β and TNFα [10].

The anti-fibrotic effect of a combination of the three ligands remains unexplored. Therefore, we investigate the effects of combined treatment including, PJ, ATRA and 9-*cis*, on key events of liver fibrosis in primary HSCs. We found that the combined treatment inhibited HSCs proliferation via cell cycle arrest and inhibition of proteins involved in cell cycle. We also found that this combination had an inhibitory effect on ECM protein expression.

## Materials and Methods

### Animals

Male retired breeder Wistar rats (300-400 g) were maintained in the animal facility of the Tel Aviv Sourasky Medical Center on a standard rat chow diet with a 12 hours light/dark cycle. The use of animals was in accordance with the NIH Policy on the care and use of laboratory animals and was approved by the Animal Use and Care Committee, the Ichilov committee.

### Isolation and culture of primary rat HSCs

HSCs were isolated using sequential pronase-collagenase perfusion followed by Nycodenz (Sigma-Aldrich, Inc., St. Louis, MO) density gradient centrifugation, as described previously [11].

### Reagents

A 10^-2^ M stock solution of PJ, ATRA and 9-*cis* (Sigma-Aldrich, Inc., St. Louis, MO) was prepared in DMSO. A stock solution of 1 µg/ml platelet-derived growth factor (PDGF-BB) (Peprotech Inc., NJ, USA) was prepared in water. TGF-β (R&D Systems Inc. MN) was dissolved in 4mM HCl containing 1mg/ml BSA at a concentration of 1 µg/ml. All stock reagents were aliquot and stored at -20°C until use.

### Calculations

The theoretical additive inhibitory affect of agents *a*, *b* and *c* was calculated as described before [12] using the following equations:

I_*ab*_ = 100 X [1 - (1-I_*a*_/100) X (1-I_*b*_/100)]

I_*ab*_ is the calculated additive inhibitory effect expressed as percent inhibition. I_*a*_ and I_*b*_ are the measured inhibitory effects (%) of each agent acting alone as compared to control. Equation 1 was derived assuming the inhibitory agents act independently on the same target population. The nature of the interaction between agents *a* and *b* was assessed by comparing the inhibitory effect of combined treatment as determined experimentally to the calculated additive inhibitory effect.

Inhibitory effect (%) = 100 X (OD_control_ – OD_treatment_) / (OD_control_ – OD_bl_)

The interaction is synergistic when the experimentally observed effect is larger than the calculated additive effect. When the experimentally observed effect is smaller than the calculated additive effect, the interaction is antagonistic, and the interaction is additive when there is no difference between the effects.

### Proliferation assay

HSCs proliferation was examined by BrdU method (Exalpha Biological, Inc. Watertown, MA). Primary HSCs were cultured for 14 days, and then 20,000 cells/well were seeded in 96 well plates. in DMEM + 10% FCS. The cells were incubated for 24h, and then medium was changed to serum starvation medium (DMEM+ 0.5% FCS) overnight. The cells were treated with various stimuli. HSCs were exposed to 30 ng/ml PDGF and either 10^-5^M PJ, 10^-5^ M ATRA, 10^-5^M 9-*cis* or combination of the three. After 24 hours the cells were tested for proliferation following the manufacturer’s instructions.

### Western blot

2x10^6^ HSCs were seeded and incubated for 10, 20, 30, 60 min or 24 hrs with different treatments according to the experiments performed. Total proteins were extracted using RIPA buffer containing protease inhibitor cocktail (Sigma-Aldrich, Inc., St. Louis, MO). Proteins were separated on 4-12% BT gels (NuPAGE, Gibco-BRL Life Technologies, Grand Island, NY) and incubated with antibodies against cyclin D1, αSMA, β-actin, GAPDH (Santa Cruz Biotechnology, Santa Cruz, CA), collagen Iα1 (Affinity Bioreagents, Golden, CO) p21, p27 (Epitomics), p-mTOR (Cell signaling), mTOR (Epitomics), p-P70^S6K^(Cell signaling), P70^S6K^ (Cell signaling).

### Oil Red O staining

Primary HSCs cultured in a 24 well plates were washed with PBS and fixed with 4% paraformaldehyde. Oil Red O in prophylene-glycol was added, washed, and lipid droplets were photographed.

### Reverse-transcription and polymerase chain reaction

1.5x10^-6^ cells/plate were incubated for 24 hrs with the different treatments according to the specific experiments. Total RNA was extracted by EZ-RNA kit (Biological industries Ltd., Israel) according to the manufacturer’s instructions. 1µg of total RNA was reversed into cDNA using High Capacity cDNA Reverse Transcription Kit (Applied Biosystems, Victoria, Australia), and analyzed using quantitative real-time PCR to determine the expression of collagen Iα1 (Rn01463848_m1) and αSMA (Rn01502596_m1). Semiquantitative RT-PCR was done using PPIA (Rn00690933_m1) as an internal control to normalize for gene expression.

### Statistical analysis

The results are presented as fold induction compared to control values, considered as being 100%, and are presented as means ±SD from at least three separate experiments. Statistical significance was assessed using Microsoft Excel software using an unpaired two tailed student t-test with P values <0.05 considered significant.

## Results

### The combination of PJ, ATRA and 9-cis is additive

To clarify the effects of combining the three ligands, PJ, ATRA and 9-*cis*, we examined the type of interaction between them. We examined the effect of the three ligands on cell proliferation and calculated the inhibitory effect. The interaction is synergistic when the experimentally observed effect is larger than the calculated additive effect. When the experimentally observed effect is smaller than the calculated additive effect, the interaction is antagonistic, and the interaction is additive when there is no difference between the two effects.

**Figure 1:**
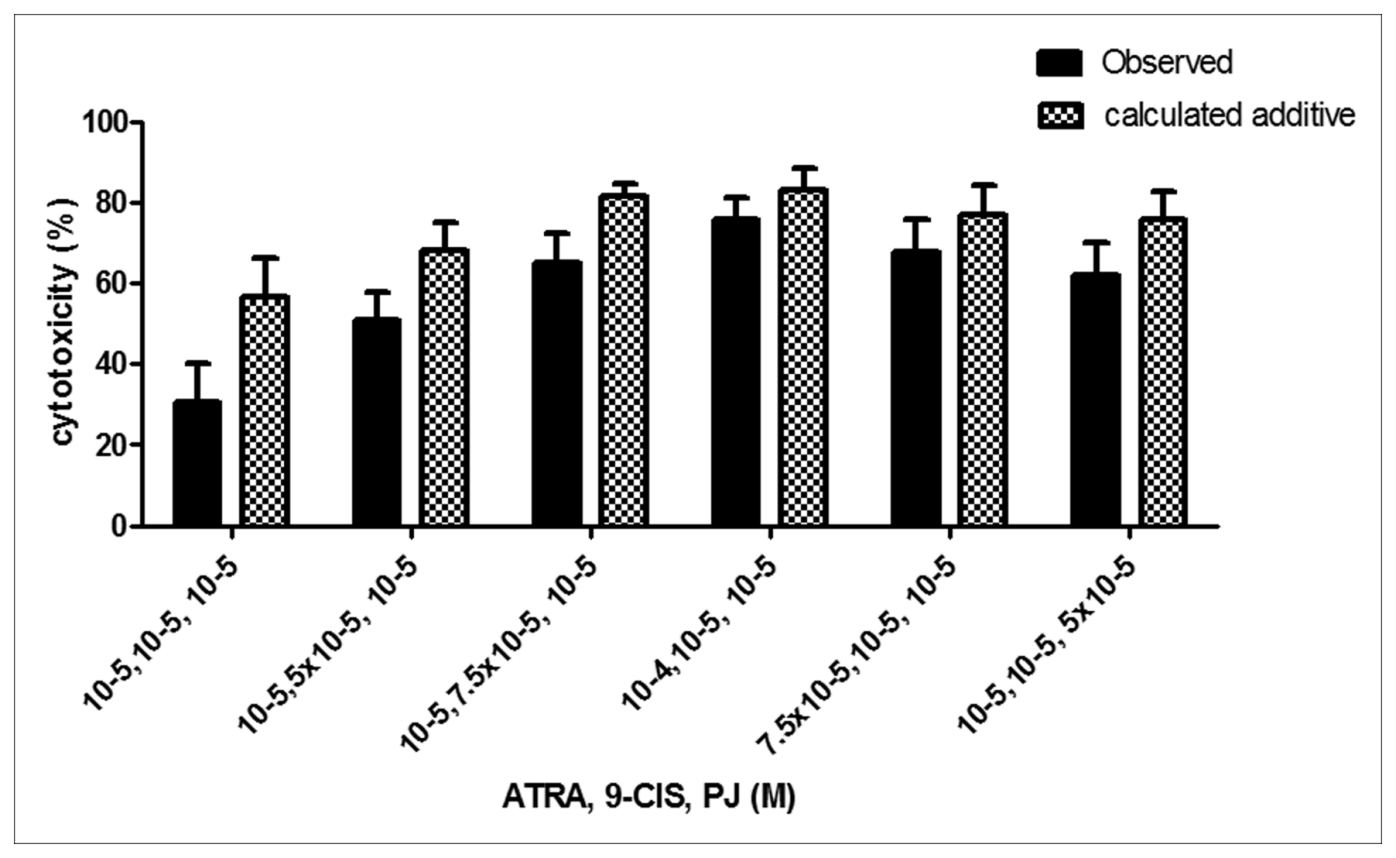
The combined anti-proliferative effect of PJ, ATRA and 9-*cis* is additive. HSCs were plated in 96 well plates. After 24 hrs, the medium was changed to starvation medium (DMEM with 0.5% FCS) overnight. HSCs were incubated for 24 hrs with 30 ng/ml PDGF, PJ, ATRA and 9-*cis* at doses of 10^-5^-10^-4^M. Cells were quantified by crystal violet staining. The inhibition of cell proliferation was expressed as % inhibition and calculated according to the following equation: Inhibitory effect (%) = 100 X (OD_control_ – OD_treatment_) / (OD_control_ – OD_bl_). The interaction is synergistic when the experimentally observed effect is larger than the calculated additive effect. When the experimentally observed effect is smaller the calculated additive effect, the interaction is antagonistic, and the interaction is additive when there is no difference between the two effects. Histogram showing average ±SE of densitometry results from 3 independent experiments.

As seen in Figure 1, the interaction between PJ, ATRA and 9-*cis* is additive.

### PJ, ATRA and 9-cis inhibited HSCs proliferation

**Figure 2:**
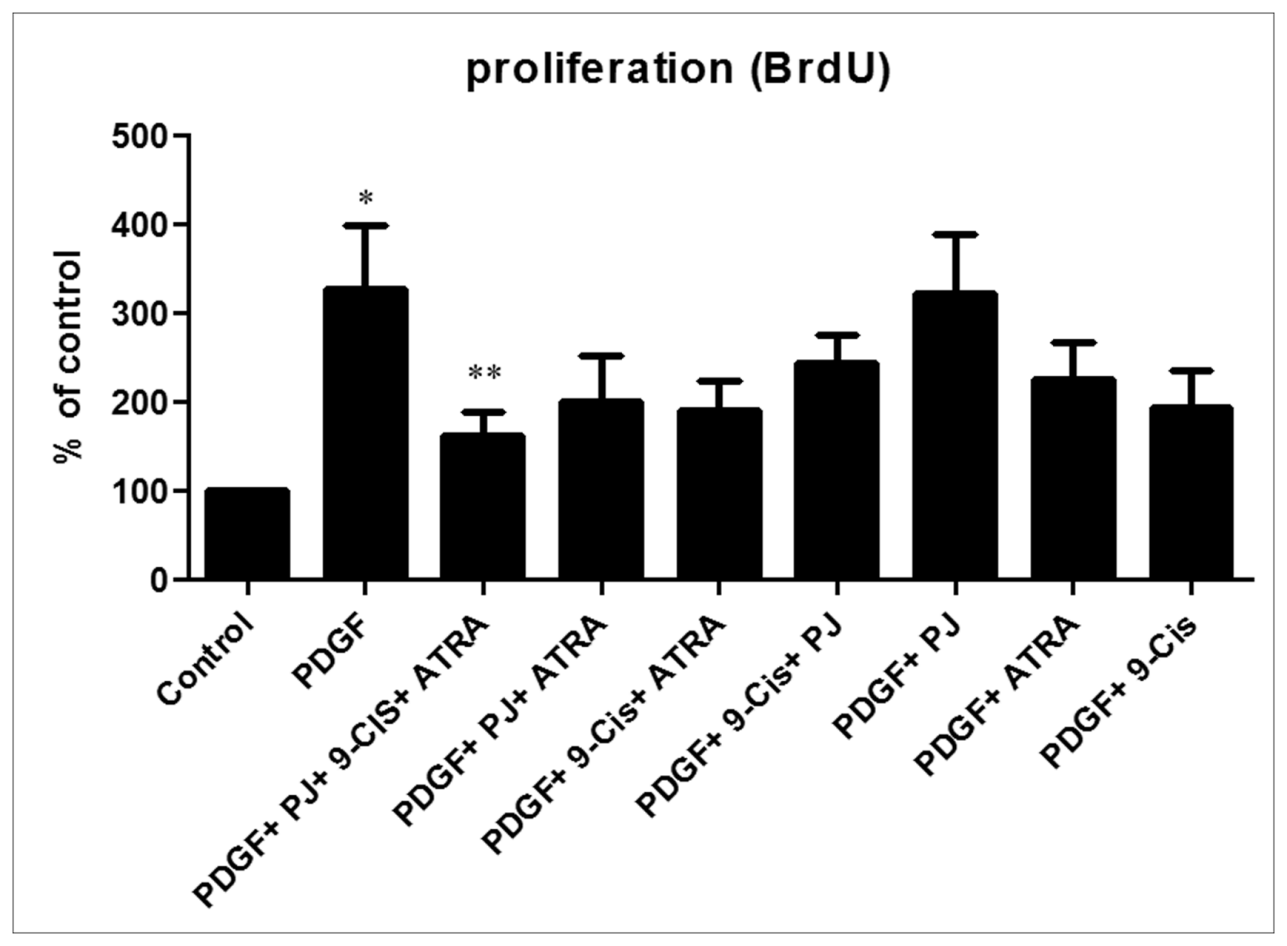
PJ, ATRA and 9-*cis* inhibit proliferation of rat primary HSCs in the presence of PDGF. HSCs were plated in 96 well plates. After 24 hrs, the medium was changed to starvation medium (DMEM with 0.5% FCS) overnight. HSCs were incubated for 24 hrs with 30 ng/ml PDGF, PJ, ATRA and 9-*cis* at a dose of 10^-5^ M. After incubation, cell proliferation was assessed using the BrdU assay and plates read using an ELISA reader at 450 nm. Histogram showing average ±SE of densitometry results from 5 independent experiments. ^*^ *P*<0.05 vs. control, ^**^ *P* <0.05 vs. PDGF.

We investigated the effect of PJ, ATRA and 9-*cis* on PDGF-induced HSCs proliferation. As expected, incubation of HSCs with PDGF induced proliferation by 3.5 fold (Figure 2). Addition of the three ligands resulted in marked inhibition of HSCs proliferation by 1.75 fold compared to PDGF treatment alone. The other combinations tested did not have the same inhibitory effect.

### Treatment with PJ, ATRA and 9-cis led to cell-cycle arrest

**Figure 3:**
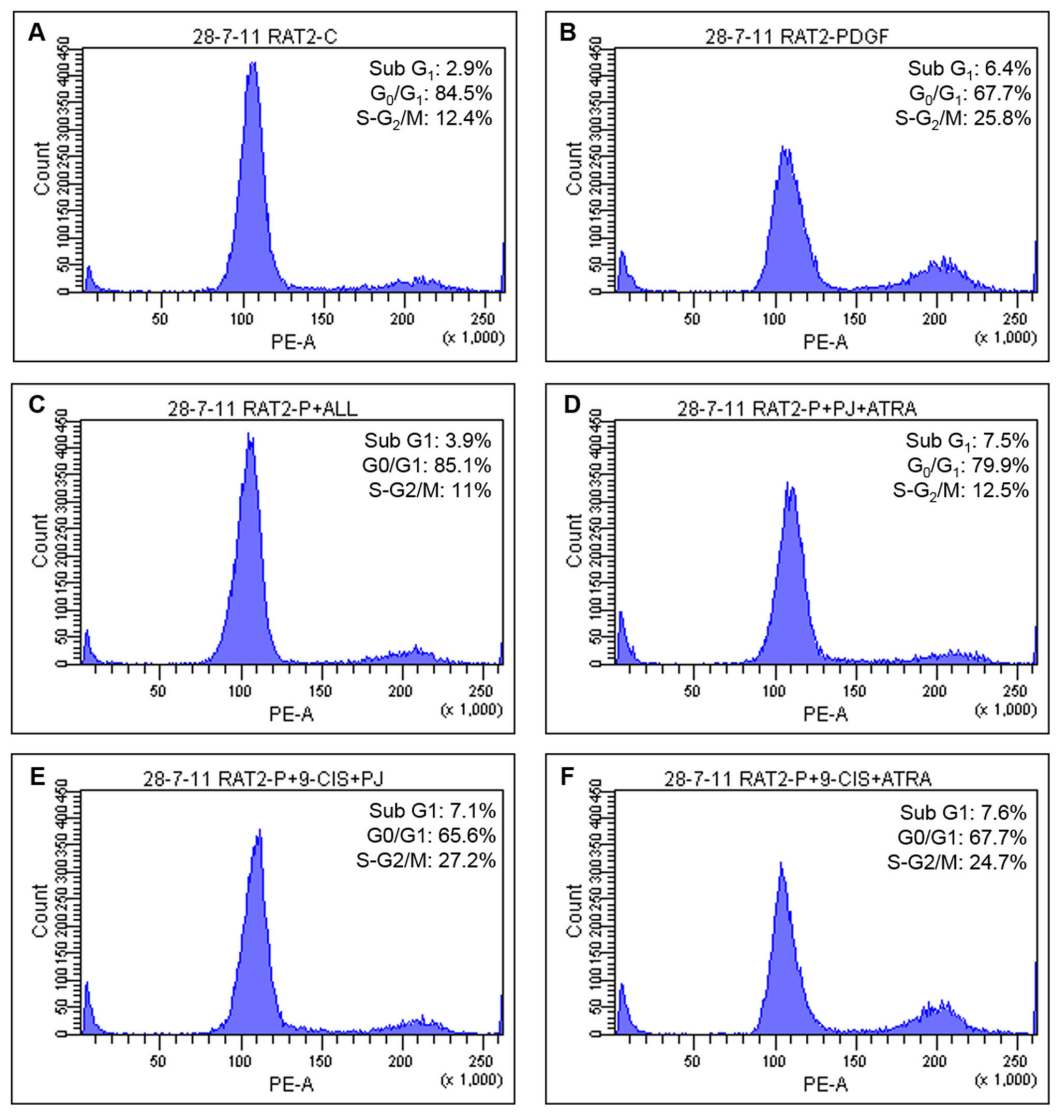
Combined treatment of PJ, ATRA and 9-*cis* led to cell cycle arrest in primary HSCs. HSCs were incubated 24 hrs with 30 ng/ml PDGF, PJ, ATRA and 9-*cis* at a dose of 10^-5^ M. Cellular DNA was stained with propidium iodide and flow cytometric analysis was preformed.

Following HSCs inhibition of proliferation, we analyzed the cell-cycle in the presence of the ligands. As expected, PDGF decreased cells number in G_0_/G_1_ phase, and increased the number of cells in S-G_2_/M phase (Figure 3B). Addition of PJ, ATRA and 9-*cis* caused cell-cycle arrest, increased the number of cells in G_0_/G_1_ phase and reduced the number of cells in the S-G_2_/M phase. Moreover, there was no increase in apoptosis as demonstrated by low sub G_1_ peak (Figure 3C). The other combinations tested had no such effect (Figure 3D,E,F). Thus, combination of the three ligands led to G_0_/G_1_ arrest.

### Effect of PJ, ATRA and 9-cis on cell-cycle proteins

**Figure 4:**
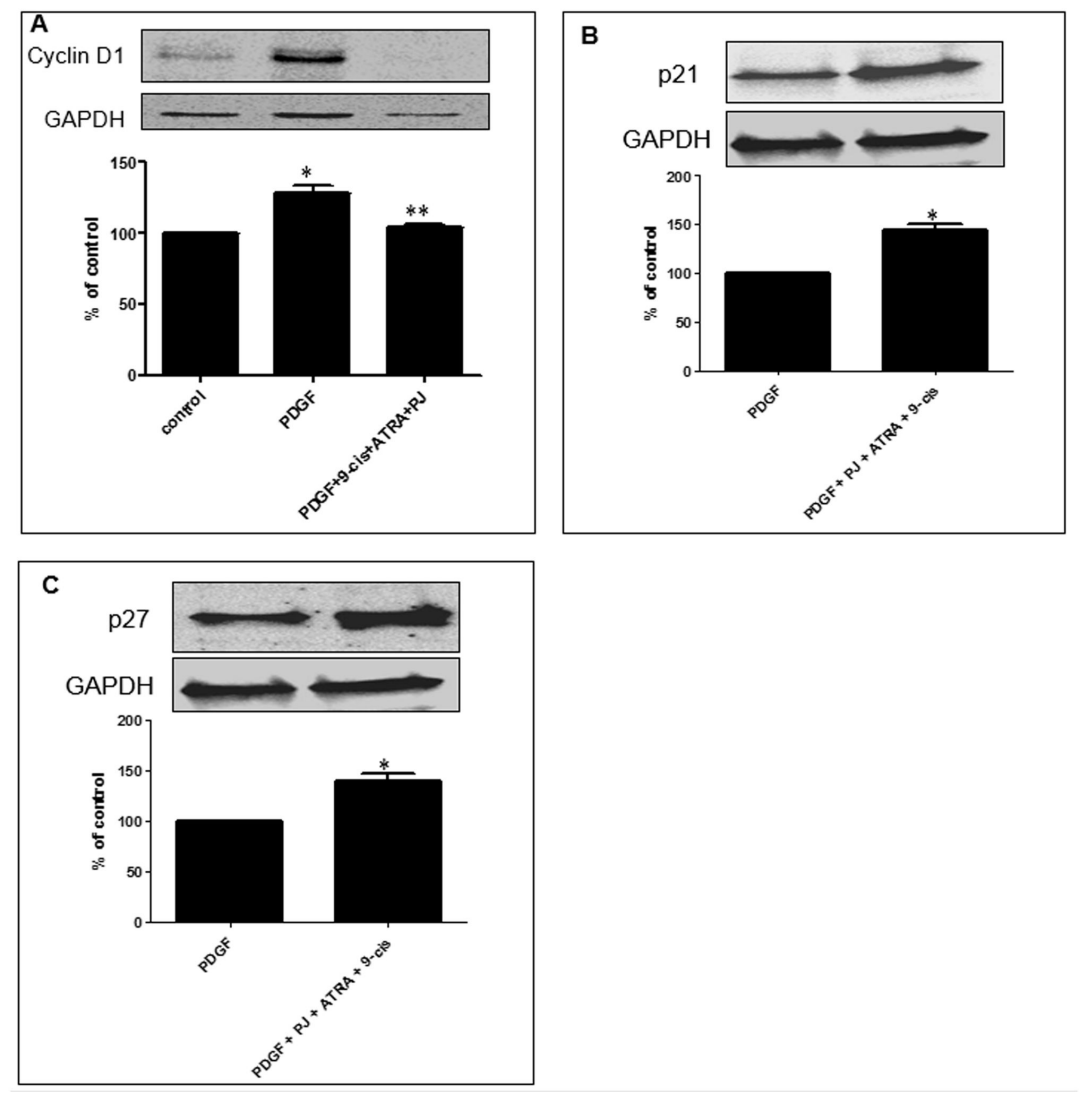
Combined treatment of PJ, ATRA and 9-*cis* effect cell cycle protein expression in primary HSCs. **A**. Combined treatment of PJ, ATRA and 9-*cis* decreased cyclin D1 expression in primary rat HSCs. **B**. Combined treatment of PJ, ATRA and 9-*cis* increased P21 expression in primary rat HSCs. **C**. Combined treatment of PJ, ATRA and 9-*cis* increased P27 expression in primary rat HSCs. HSCs were incubated for 24 hrs with 30 ng/ml PDGF, PJ, ATRA and 9-*cis* at a dose of 10^-5^ M. Total lysate was separated by western blot analysis. Histogram showing average ±SE of densitometry results from 3 independent experiments. ^*^ *P*<0.05 vs. PDGF.

We assessed the effects of the combined treatment on proteins regulates the cell-cycle. We found that treatment with the three ligands suppressed cyclin D1 expression to the control levels (Figure 4A).

Determination of p21 and p27 proteins, which are inhibitors of the cell-cycle, showed an increased expression (by ~50%) in the presence of the three ligands (Figure 4C,D). These results support the hypothesis that the combined treatment of PJ, ATRA and 9-*cis* lead to cell cycle arrest.

### Effect of PJ, ATRA and 9-cis on PDGF’s signaling

**Figure 5:**
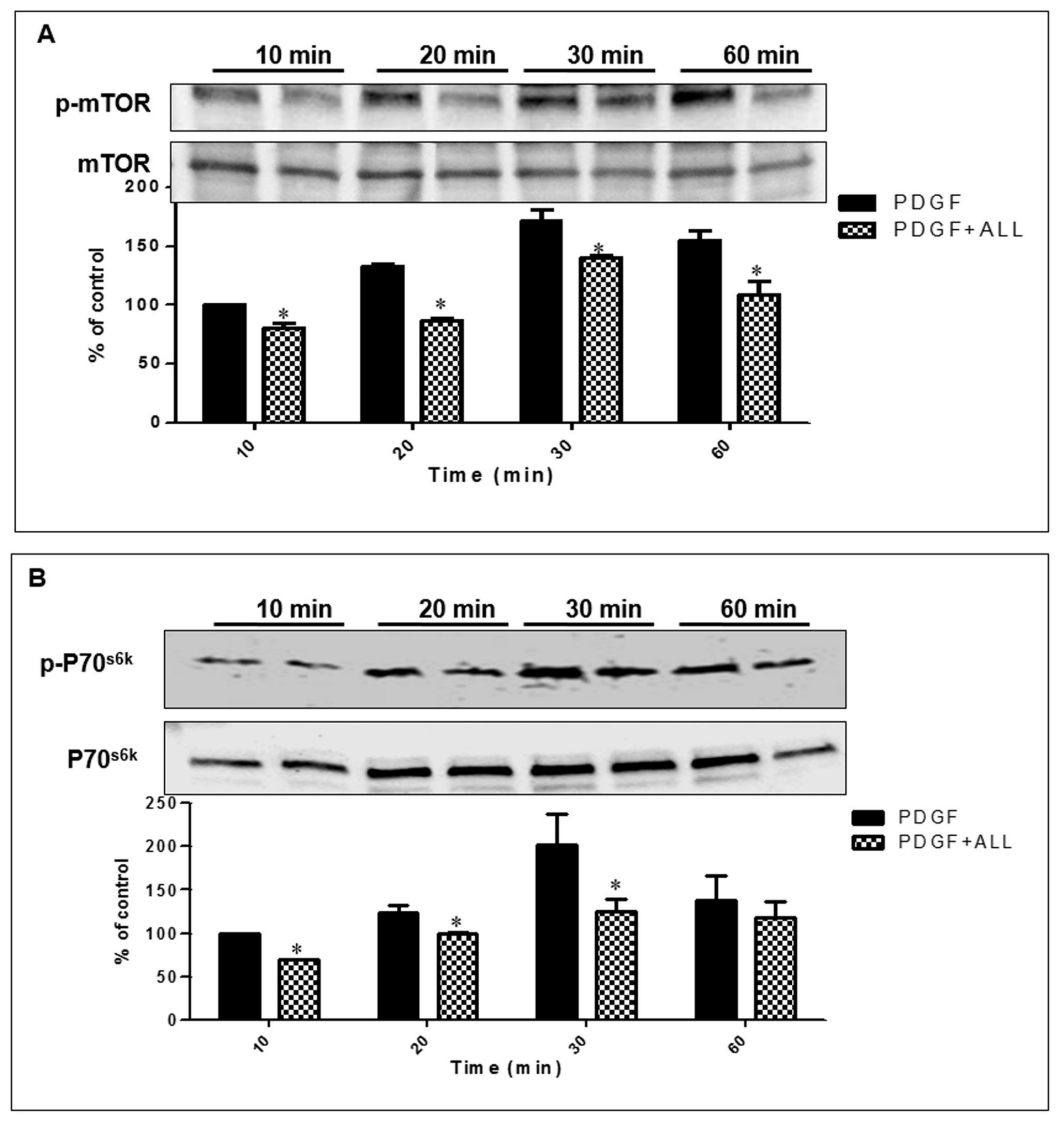
Combined treatment of PJ, ATRA and 9-*cis* inhibit mTOR. (A) and p70^S6K^ (B) phosphorylation in primary HSCs. HSCs were incubated for 10, 20, 30 and 60 mins with 30 ng/ml PDGF, PJ, ATRA and 9-*cis* at a dose of 10^-5^ M. Total lysate was separated by western blot analysis. Histogram showing average ±SE of densitometry results from 3 independent experiments. ^*^ *P*<0.05 vs. PDGF.

We next investigated whether the effects of PJ, ATRA and 9-*cis* are mediated through mTOR signaling pathway. We observed that exposure to PDGF resulted in mTOR phosphorylation in a time dependent manner (Figure 5A) and incubation with PJ, ATRA and 9-*cis* inhibited this phosphorylation.

We next determined the effect of PJ, ATRA and 9-*cis* on p70^S6K^ which is downstream to mTOR. Treatment with the three ligands decreased p70^S6K^ phosphorylation levels (Figure 5B). These results indicate that the combination PJ, ATRA and 9-*cis* suppresses mTOR signaling.

### Effect of PJ, ATRA and 9-cis on HSCs content of lipid droplets

**Figure 6:**
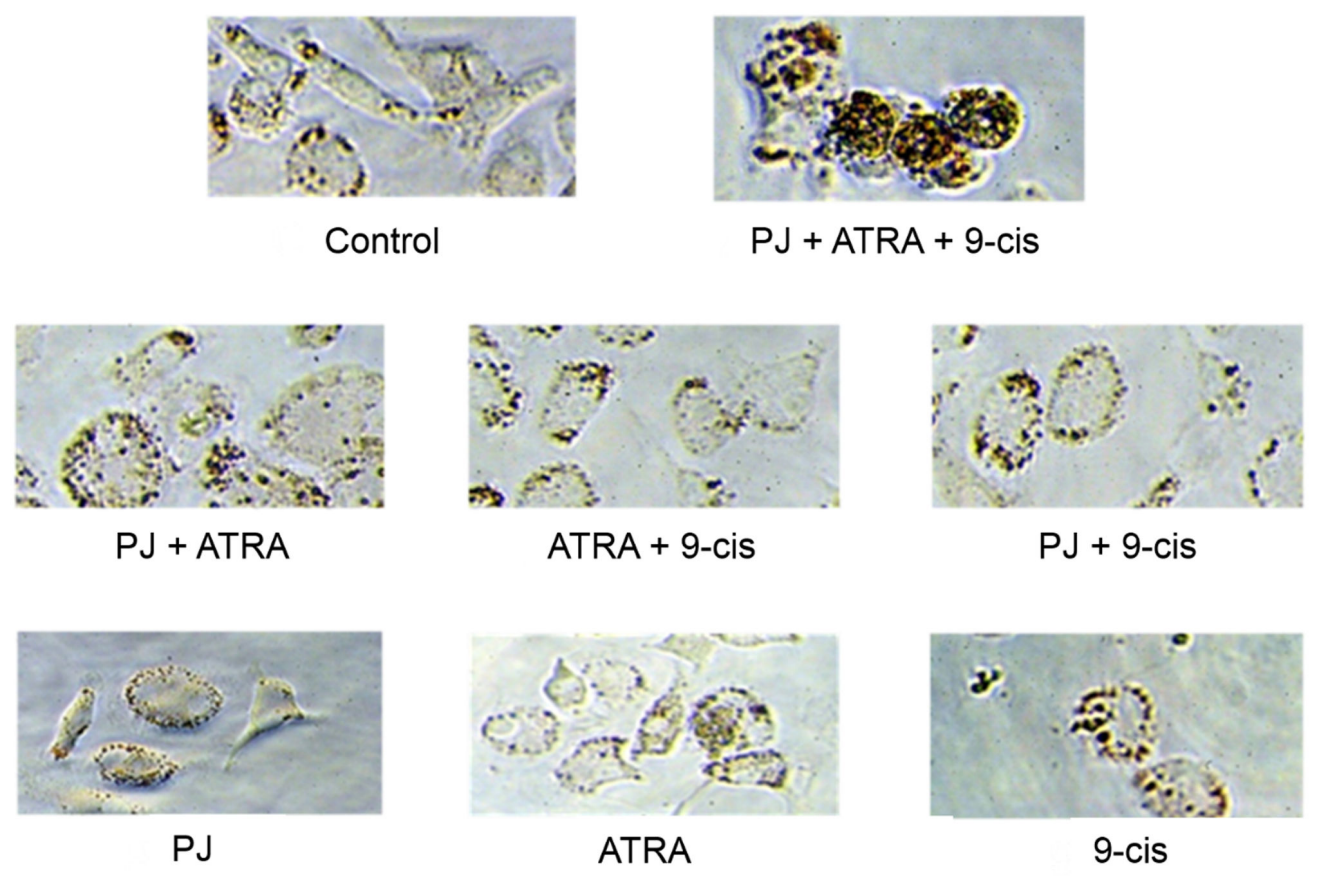
Combined treatment of PJ, ATRA and 9-*cis* inhibit lipid droplets release in primary HSCs. HSCs were plated in 96 well plates. After 5 days, the medium was changed to starvation medium (DMEM with 0.5% FCS) overnight. The next day, PJ, ATRA and 9-*cis* were added at a dose of 10^-5^ M. After incubation for 24 hrs, lipid content was assessed using the Oil red O assay.

A characteristic feature of HSCs activation is the loss of the retinoid-containing lipid droplets [13]. To investigate whether PJ, ATRA and 9-*cis* effect the release of the lipid droplets, we examined the HSCs lipid droplets contents. As shown in Figure 6, the untreated cells showed low lipid contents located at the edges of the cells. On the other hand, HSCs incubated with the three ligands contained significantly more lipid droplets that were distributed in the cytoplasm. These results indicate that only the combined treatment led to inhibition of lipid droplets release.

### Effect of PJ, ATRA and 9-*cis* on fibrotic markers

**Figure 7:**
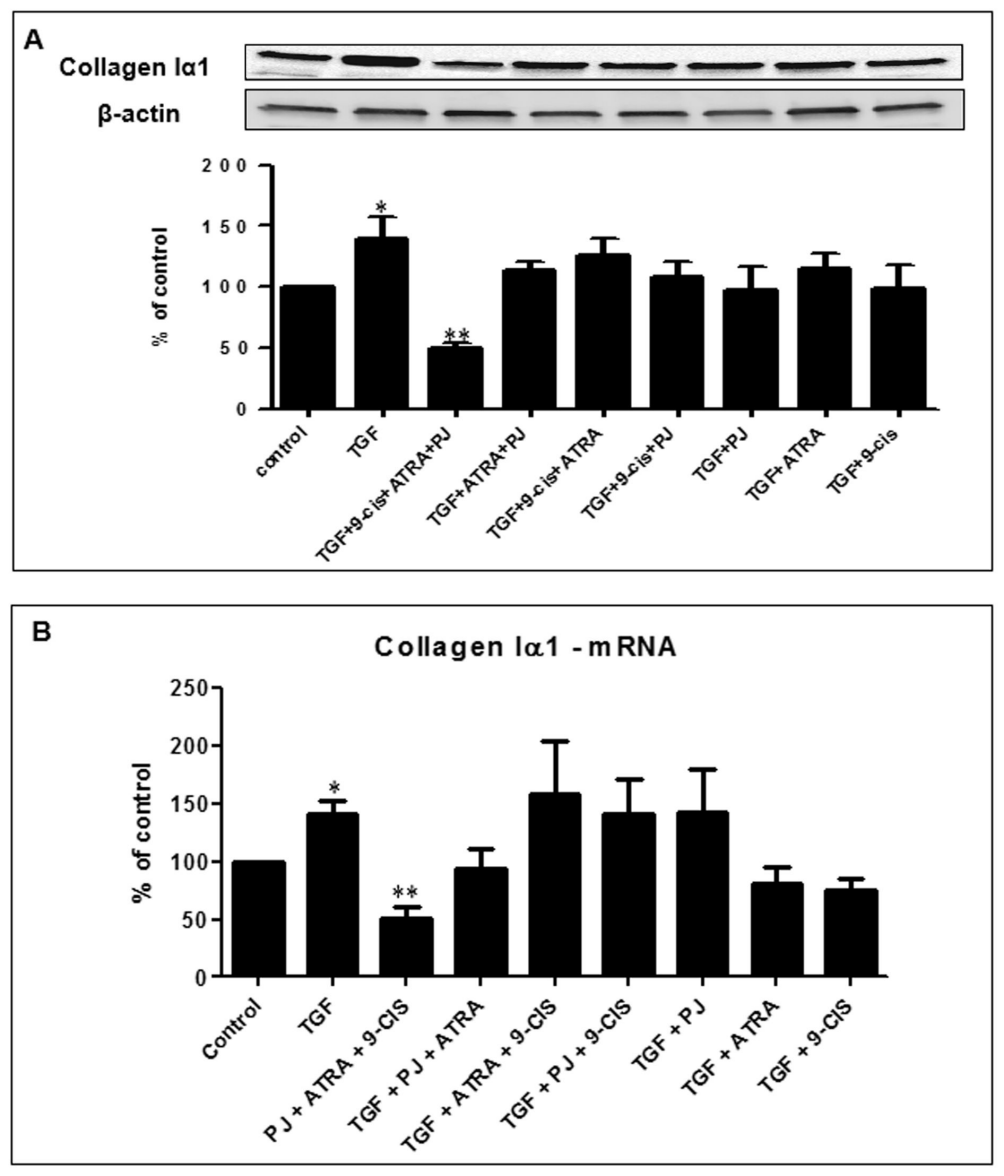
Suppression of collagen Iα1 protein expression and mRNA by combined treatment of PJ, ATRA and 9-*cis*. Western blot analysis of primary cultured HSCs that were incubated for 24 hrs with 10 ng/ml TGF-β, PJ, ATRA and 9-*cis* at a concentration of 10^-5^ M (A). Total RNA was isolated from HSCs treated overnight with 10 ng/ml TGF-β, PJ, ATRA and 9-*cis* at a concentration of 10^-5^ M and analyzed by quantitative real-time PCR using primers specific to collagen Iα1. The results were normalized to β-actin mRNA expression levels. Data are expressed as mean + SE ^*^ *p*<0.05 vs. TGF-β (B).

To investigate whether PJ, ATRA and 9-*cis* has an antifibrotic effect on HSCs, collagen Iα1 expression was examined. TGF-β increased collagen Iα1 protein expression by ~50%, while the combined treatment of the three ligands suppressed collagen Iα1 expression by ~33% compared to TGF-β (Figure 7A). Similar results were obtained at the mRNA level (Figure 7B).

**Figure 8:**
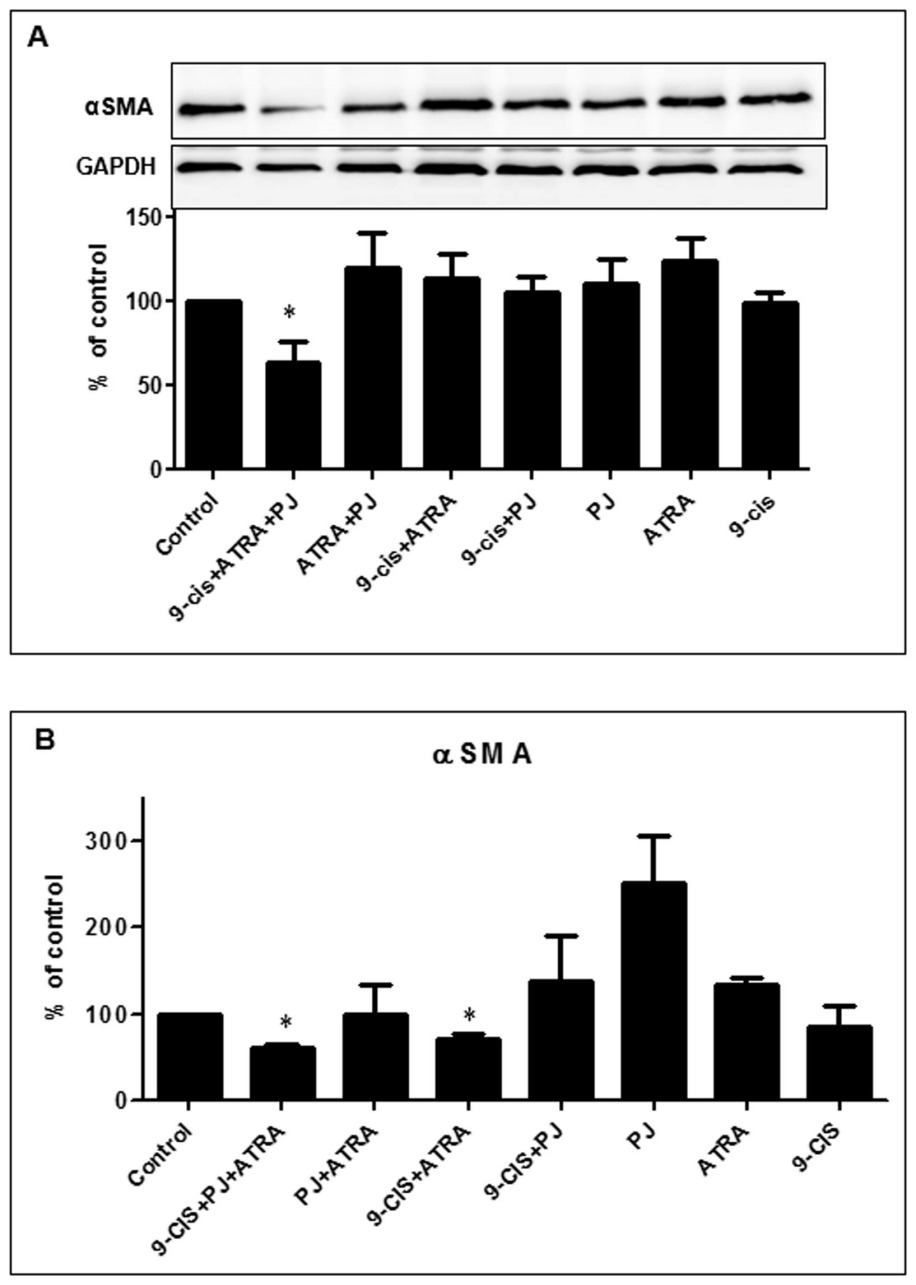
Suppression of αSMA protein expression and mRNA by combined treatment of PJ, ATRA and 9-*cis*. Western blot analysis of primary cultured HSCs that were incubated for 24 hrs with PJ, ATRA and 9-*cis* at a concentration of 10^-5^ M (A). Total RNA was isolated from HSCs treated overnight with PJ, ATRA and 9-*cis* at a concentration of 10^-5^ M and analyzed by quantitative real-time PCR using primers specific to αSMA. The results were normalized to β-actin mRNA expression levels. Data are expressed as mean + SE ^*^ *p*<0.05 vs. control (B).

When we examined the expression of αSMA, another marker of HSCs activation, we found that the combined treatment of the three ligands decreased αSMA protein levels by ~40% but no effect was observed with each of the ligands alone (Figure 8A). Similar reduction was obtained on αSMA mRNA levels (Figure 8B).

## Discussion

Nuclear receptors control a large variety of metabolic pathways including hepatic lipids metabolism, inflammation, fibrosis, cell differentiation and tissue repair including liver regeneration [14,15].

The present study was undertaken to investigate the interaction between PJ, ATRA and 9-*cis* and especially their potential anti-fibrotic effects in rat primary HSCs, since each of the ligands alone showed only a modest effect on liver fibrosis in HSCs as well as *in vivo* models [3,4,9,16].

We showed that exposure of HSCs to combined treatment of the three ligands resulted in significant inhibition of cell proliferation. Interestingly, combined treatment with only two ligands did not lead to such a reduction in proliferation. In our previous study, we found that combined treatment of 9-*cis* and PJ or ATRA and PJ also decreased HSCs cell line proliferation [10].

In this study, we found that the effect of the three ligands on HSCs proliferation was additive. Previous studies showed that ligands of PPARγ, RAR and RXR had similar effects in other cell lines. Combined treatment of ligands of PPARγ and RAR led to proliferation inhibition, cell-cycle arrest and increased PTEN expression in HL-60 cells [17]. PPARγ and RAR ligands inhibited human breast cancer cells proliferation, cell invasion, MMP-9 activity and up regulation of TIMP-1 expression [16]. Combination of PPARγ and RAR ligands led to cell-cycle arrest in human glioblastoma cell line [18]. In addition, PPARγ can bind to the RARE as a PPARγ-RXR heterodimer and regulated its expression in lung and breast cancer cell line [19].

PJ, ATRA and 9-*cis* can lead to cell-cycle arrest in many cell types [3,16,20-23]. In pancreatic cancer cell line, 9-*cis* together with troglitazone (a PPARγ ligand) decreased proliferation and led to cell-cycle arrest in G_0_/G_1_ phase [24,25]. In the present study, we observed cell-cycle arrest associated with an increase in G_0_/G_1_ and decrease in S phase in HSCs treated with PJ, ATRA and 9-*cis*, but not with other combinations of ligands. We also found down-regulation of cyclin D1 and up-regulation of p21 and p27 protein levels in HSCs that were treated with the three ligands. These results are supported by previous studies showing that PPARγ ligands can down-regulate the expression of cyclin D1 [26] as well as up-regulate the expression of p21 [27]. ATRA down-regulated cyclin D1 expression [28,29] and induced p27 [30]. In addition, combined treatment of 9-*cis* and troglitazone, decreased cyclin D1 expression in pancreatic cancer (20).

One of the critical pathways to proliferation and survival in many cell types, including HSCs [31] is the mTOR/p70^S6K^. When studied the signaling pathway of mTOR and p70^S6K^ in HSCs, we found that PJ, ATRA and 9-*cis* reduced phosphorylation of mTOR and p70^S6K^, leading to interruption of the mitogenic PDGF signaling pathways. These results suggest that PJ, ATRA and 9-*cis* might mediate the effect of mTOR/p70^S6K^ signaling pathways on HSCs growth inhibition. Our findings are consistent with Lee et al studies demonstrating that combined administration of 9-*cis* and PJ to hepatocytes led to down-regulation of mTOR and p70^S6K^, resulting in TGF-β inhibition [26].

Under physiological conditions, HSCs store about 80% of the total body content of vitamin A in lipid droplets and play a pivotal role in the regulation of vitamin A homeostasis [32]. Activation of cultured HSCs correlates with depletion of vitamin A droplets [33]. In our study, the only treatment that prevented the depletion of the lipid droplets was the combined treatment of PJ, ATRA and 9-*cis*.

We also examined the effect of the combined treatment of PJ, ATRA and 9-*cis* on fibrosis markers, i.e. collagen Iα1 and αSMA. The combined treatment of the three ligands significantly inhibited the expression of these fibrosis markers at the protein and the mRNA levels. PJ and ATRA are known to down regulate fibrotic markers while 9-*cis* can increase procollagen I mRNA but has no effect on other matrix proteins [6,8]. In a previous study, we showed that combined treatment of ATRA and rosiglitazone reduced αSMA and collagen content in a rat model of liver fibrosis [10].

Taken together, our results suggest that combination of the three ligands PJ, ATRA and 9-*cis* caused inhibition of HSCs proliferation by cell cycle arrest and down-regulation of fibrotic markers to a much greater extent compared to each of the ligands alone. Additional studies may be necessary to reveal the exact mechanism(s) responsible to the vigorous inhibitory effect of the three ligands on HSCs proliferation compared to each agent alone.
